# Health-related quality of life of men with primary osteoporosis and its changes after bisphosphonates treatment

**DOI:** 10.1186/s12891-023-06397-8

**Published:** 2023-04-19

**Authors:** Di-chen Zhao, Xiao-yun Lin, Jing Hu, Bing-na Zhou, Qian Zhang, Ou Wang, Yan Jiang, Wei-bo Xia, Xiao-ping Xing, Mei Li

**Affiliations:** Department of Endocrinology, National Health Commission Key Laboratory of Endocrinology, Peking Union Medical College Hospital, Chinese Academy of Medical Sciences and Peking Union Medical College, Shuaifuyuan No. 1, Beijing, Dongcheng District 100730 China

**Keywords:** Osteoporosis, Males, HRQoL, SF-36, Bisphosphonates

## Abstract

**Introduction:**

Osteoporosis leads to more serious consequences in men than in women, but less is known about its impacts on health-related quality of life (HRQoL) of men, and whether the anti-osteoporosis treatment can improve HRQoL of men with osteopenia/osteoprosis.

**Methods:**

We enrolled men with primary osteoporosis and age-matched healthy controls. We collected medical history, serum levels of carboxyl-terminal type I collagen telopeptide, procollagen type I propeptides, and bone mineral density of patients. All patients and controls completed the short-form 36 (SF-36) questionnaires. Changes in HRQoL of osteopenia/osteoporosis men were prospectively evaluated after alendronate or zoledronic acid treatment.

**Results:**

A total of 100 men with primary osteoporosis or osteopenia and 100 healthy men were included. The patients were divided into three subgroups: osteopenia (n = 35), osteoporosis (n = 39) and severe osteoporosis (n = 26). Men with osteoporosis or severe osteoporosis had impaired HRQoL in domains of physical health compared to healthy controls. HRQoL scores in physical health related domains of patients with severe osteoporosis were significantly lower compared to healthy controls, and were the poorest among the three subgroups of patients. Fragility fracture history was correlated with lower SF-36 scores about physical health. In 34 men with newly diagnosed osteoporosis receiving bisphosphonates treatment, HRQoL scores were significantly improved in domains of physical health after treatments.

**Conclusions:**

The HRQoL is significantly impaired in men with osteoporosis, and the more severe the osteoporosis, the poorer the HRQoL. Fragility fracture is an important influencing factor of deteriorated HRQoL. Bisphosphonates treatment is beneficial to improve HRQoL of osteopenia/osteoporosis men.

**Supplementary Information:**

The online version contains supplementary material available at 10.1186/s12891-023-06397-8.

## Introduction

Osteoporosis is a progressive disease leading to low bone mineral density (BMD), impaired bone strength and increased bone fracture risk [1]. Osteoporosis has been gradually recognized as a major health problem in men in recent years, as it is estimated that 6.6% and 6.0% of men suffered from osteoporosis in Europe and China [2–4]. Osteoporosis causes more than 2 million fractures per year in the US, of which 29% fracture occurred in men [5]. In China, the prevalence of vertebral fracture was 10.5% among men and 9.7% among women [3]. Meanwhile, men tend to have more complications and higher mortality after osteoporotic fracture than women [6]. Hip fractures can give rise to limited mobility, high risk of deep venous thrombosis, even cardiovascular and cerebrovascular events, which lead to 2–3 times more mortality rates in men than in women [7]. Although the prevalence of osteoporosis and osteoporotic fracture is not so low in men, and the consequences are even more severe than those in women, male osteoporosis is still under-screened, underdiagnosed and undertreated [5].

As we know, the most serious consequence of osteoporosis is fragility fractures, and poor health-related quality of life (HRQoL) and impaired physical function are closely associated to vertebral fractures, non-vertebral fractures and hip fractures in postmenopausal women with osteoporosis [8, 9]. HRQoL is concerned with health aspects such as physical, emotional and social wellbeing, and with the effect of illness and treatment on these parameters [10]. One of the most widely used generic questionnaires to quantify HRQoL is the 36-Item Short Form Health Survey (SF-36), which is validated for use in women with osteoporosis [11]. Most of the studies concerning HRQoL in patients with osteoporosis are completed in either exclusive female samples [8] or mixed male and female samples [12, 13], and the influences of osteoporosis on HRQoL are rarely reported in relatively large cohort of men. A recent meta-analysis reveals that HRQoL is impaired in men with osteoporosis, but it is based on a limited number of heterogenous studies [14]. Moreover, risk factors for impaired HRQoL are unclear in men with osteoporosis, whether HRQoL is related to low BMD or positive history of fracture deserves to be studied in men.

As we know, the treatment of osteoporosis has made great progress. Depending on the mechanism of action, anti-osteoporotic agents are mainly divided into four categories: essential medicines, bone resorption inhibitors, bone formation promoters and dual acting drugs [15]. Among them, bisphosphonates, denosumab and teriparatide are widely used therapeutic drugs for osteoporosis in men, which can effectively increase BMD, and reduce the risk of bone fractures [16–19]. However, the effects of anti-osteoporotic agents on HRQoL of men with osteoporosis is unclear.

Therefore, we aim to investigate the HRQoL and its influencing factors, and to prospectively observe its changes in a cohort of men with primary osteoporosis or osteopenia who receive bisphosphonates treatment.

## Subjects and methods

### Study population

This study was conducted from July 2018 to July 2022 in the Endocrinology Department of Peking Union Medical College Hospital (PUMCH). The study was approved by the scientific ethic committee of PUMCH (JS-2798). Informed consents were obtained from all patients and healthy controls before they participated in this study.

Men who visited the Endocrinology Department of PUMCH and with chief complaints of bone pain or a history of fragility fracture were suspected as osteoporosis, and were recruited by endocrinologists. Clinical information was collected in detail, including age, history of fragility fracture, history of anti-osteoporosis treatments, comorbidities, family history of fragility fracture, skeletal deformity, mobility, etc. Height and weight of patients were measured with a Harpenden stadiometer (Seritex Inc). Body mass index (BMI) was calculated as weight in kilograms divided by height in meters squared.

### Inclusion/exclusion criteria

Patients were eligible for inclusion if they had a BMD T-score of − 1.0 or less if the patients were more than 50 years old, or a BMD Z-score of -2.0 or less for patients less than 50 years old, with or without a history of fragility fractures. Fragility fractures were defined as fractures occurring with less than or equivalent force as a fall from standing height [20]. Exclusion criteria were as follows: (1) with other metabolic or genetic bone diseases, such as primary hyperparathyroidism, osteomalacia, Paget's disease, osteogenesis imperfecta, and so on; (2) with secondary osteoporosis, such as primary or secondary hypogonadism, celiac disease, long-term immobility, epilepsy treated with antiepileptic drugs, autoimmune diseases with treatment history of glucocorticoid, and so on; (3) with malignancy disease, such as prostate cancer, pulmonary carcinoma, hepatocarcinoma, and so on; (4) with other severe diseases that could affect HRQoL; (5) with contradictions for bisphosphonates, including renal insufficiency, severe hepatic dysfunction, or an allergy to bisphosphonates.

According to the World Health Organization (WHO)-criteria, men were classified as osteopenia, osteoporosis or severe osteoporosis. For patients who were or over 50 years old: BMD T-score of -1.0 to -2.5 as osteopenia, BMD T-score less than or equal to -2.5 as osteoporosis, BMD T-score less than or equal to -2.5 and with history of fragility fractures as severe osteoporosis. For patients who were younger than 50 years old: BMD Z-score less than or equal to -2.0 as osteoporosis, BMD Z-score less than or equal to -2.0 and with history of fragility fractures as severe osteoporosis [20, 21].

We also included 100 age-matched healthy Chinese men as a control group, who came to the outpatient department of PUMCH for healthy examinations, and without symptoms related to bone diseases (eg, bone pain, fragility fracture, kyphosis, bone deformities, immobility), history of any comorbidities or treatment that may influence the bone health (eg, hyperparathyroidism, antiviral drugs, and so on), history or evidence of psychological condition, and history of cancer.

### Evaluation of HRQoL

HRQoL was assessed by simplified Chinese version of short-form 36 (SF-36). SF-36 is a widely used questionnaire to assess the HRQoL of general and specific adult populations, estimate the relative burden of different diseases, and examine the impact of various of treatment interventions on HRQoL, and is considered valid for use in osteoporosis [22–24]. We therefore could compare the HRQoL of osteopenia/osteoporosis men to healthy controls using the same scale [25].

The SF-36 was a self-assessment of QoL over the previous four weeks, which had eight domains of health, including physical functioning (PF), role-physical limitation (RP), bodily pain (BP), general health (GH), vitality (VT), social functioning (SF), role-emotional limitation (RE), and mental health (MH). In each domain, the score ranged from 0 to 100, with higher scores indicating less pain or better functioning. Domains of PF, RP, BP and GH could be merged in a comprehensive index for physical functioning (physical component summary, PCS), as well as VT, SF, RE and MH could compose a comprehensive index for mental functioning (mental component summary, MCS) [26]. All patients completed the questionnaires of SF-36 by themselves at the hospital without help from healthy practitioners. All healthy controls also completed the questionnaires of SF-36 independently.

### Measurement of biochemical parameters and BMD of patients with osteopenia or osteoporosis

Venous blood samples were obtained after fasting for at least 8 h. Serum levels of calcium (Ca), phosphorus (P), alkaline phosphatase (ALP, a bone formation marker), alanine aminotransferase (ALT), creatinine (Cr) were measured using an automatic biochemical analyzer (Cobas Intergra 400 plus, Roche kit). Serum concentrations of luteinizing hormone (LH), follicle-stimulating hormone (FSH), testosterone (T), carboxyl-terminal type I collagen telopeptide (β-CTX, a bone resorption marker), procollagen type I propeptides (PINP, a bone formation marker), 25-hydroxy-vitamin D (25OHD) and parathyroid hormone (PTH) were detected using an automated electrochemiluminescence system (Roche Diagnostics, Switzerland). To exclude secondary osteoporosis and other metabolic bone diseases, serum levels of cortisol, adrenocorticotropic hormone, thyroid hormone, immunofixation electrophoresis were detected. All biochemical parameters were measured by the central laboratory of PUMCH.

Thoracolumbar spine X-rays films were examined, and vertebral compression fractures (VCFs) were diagnosed by radiologist using the Genant's semi-quantitative method [27]. The areal BMD at lumbar spines 1–4 (L1-4), femoral neck (FN), and total hip (TH) was measured by dual energy X-ray absorptiometry (DXA, GE Lunar Prodigy) in the radiology department of PUMCH. The coefficients of variation of the DXA measurements were 1.1%, 1.7%, and 1.1% at LS, FN, and TH, respectively.

### Anti-osteoporosis treatments and follow-up

There were 34 patients newly diagnosed as osteoporosis or osteopenia, and started to receive alendronate (ALN) or zoledronic acid (ZOL) treatment at the baseline. The other 66 patients had previous treatment history of ALN or ZOL treatment before the enrollment. ALN (Fosamax; Merck Sharp & Dohme Ltd) was taken by 70 mg with at least 250 ml of water weekly. Intravenous ZOL (Aclasta; Novartis Pharma Schweiz AG) was infused at a dose of 5 mg annually. All 100 patients were supplemented with 600 mg of calcium and 125 IU of vitamin D3 (Caltrate D; Wyeth Pharmaceuticals) daily.

Patients were asked to revisit the outpatient department every 3 months, and SF-36 surveys were collected every 3 months. Serum levels of Ca, P, ALP, PINP, β-CTX, PTH and 25OHD were detected every 3 months, and BMD was measured every 6 months.

### Statistical analysis

Continuous data following a normal distribution (including age, BMI, BMD, serum levels of biochemical parameters, baseline SF-36 domain scores) were presented as the mean ± standard deviation. Categorical data (including fragility fracture history, anti-osteoporosis treatments history) were expressed as numbers and percentages. Group differences in dichotomous variables were tested for significance using the chi-square test. Comparison of continuous variables between total patients and healthy controls was completed by two independent-sample *t* test. Differences of SF-36 among the three subgroups of patients and the control group were analyzed using one-way analyses of variance (ANOVA), and results were also adjusted for age by one-way analysis of covariance (ANCOVA). A multiple regression analysis was applied to evaluate the influencing factors (including age, time since diagnosis, BMI, history of fragility fracture, levels of T, 25(OH)D, PINP, β-CTX, and BMD at L1-4) of baseline HRQoL in patients with osteopenia, osteoporosis, or severe osteoporosis.

For patients who started to receive bisphosphonates treatment at the baseline, a paired-samples *t* test was used to longitudinally compare the differences in continuous variables (including change of HRQoL, BMD, and bone metabolic parameters) between baseline and the last visit. Multiple regression analysis was used to investigate the respective correlation between changes of BMD, bone metabolic markers and HRQoL scores, which adjusted for age and fragility fracture history.

All tests were two-tailed and a *P* value less than 0.05 was considered as statistical significance. Statistical analyses were performed using SPSS software of version 26.0 (SPSS Inc., Chicago, IL, USA).

## Results

### Baseline characteristics of the patients

**Fig. 1:**
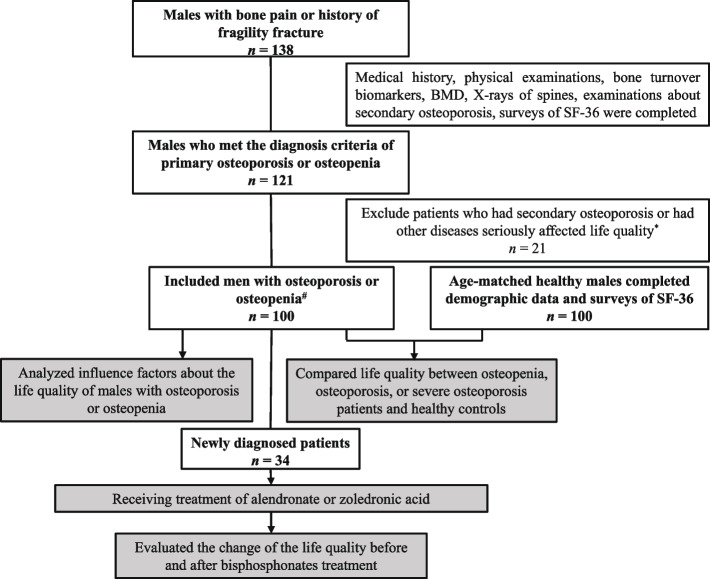
Flow chart of the study procedure

The study procedure was shown in Fig. 1, and a total of 100 patients with primary osteopenia (n = 35), or osteoporosis (n = 39) or severe osteoporosis (n = 26), and 100 age-matched healthy men were included in the analysis. The general characteristics of patients and healthy controls were shown in Table 1.

**Table 1:** Baseline characteristics of men with osteoporosis and healthy controls

	**Osteopenia**	**Osteoporosis**	**Severe osteoporosis**	**Total patients**	**Controls**
Numbers	35	39	26	100	100
Age (± SD), year	62.7 ± 8.9^***^	45.0 ± 12.9^*, ###^	56.9 ± 14.3^^^^	54.3 ± 14.4	53.1 ± 14.3
Age of diagnosis (± SD), year	60.2 ± 7.7	42.7 ± 12.6^###^	53.2 ± 12.1^^^	51.5 ± 14.0	/
Height (± SD), cm	1.72 ± 0.06	1.75 ± 0.06	1.70 ± 0.07^^^	1.73 ± 0.07	1.73 ± 0.12
Weight (± SD), kg	69.40 ± 9.44	69.15 ± 10.83	71.95 ± 11.75	69.97 ± 10.58	71.08 ± 10.13
BMI (± SD), kg/m^2^	23.43 ± 2.75	22.61 ± 3.44	24.78 ± 3.59	23.46 ± 3.34	23.77 ± 3.29
Type 2 diabetes, n (%)	12 (34.3)^*^	4 (10.3)^#^	4 (15.4)	20 (20.0)^**^	8 (8.0)
Fragility fracture, n (%)	0 (0.0)	0 (0.0)	26 (100.0)^*, #,^ ^	26 (26.0)^***^	0 (0.0)
Treatments, n (%)	19 (54.3)^*^	27 (69.2)^*^	20 (76.9)^*^	66 (66.0)^***^	0 (0.0)
Alendronate sodium, n	16	18	9	43	0
Zoledronic acid, n	3	9	11	23	0
Laboratory indexes (± SD)					
Luteinizing hormone, IU/L	3.42 ± 1.25	3.92 ± 2.13	5.88 ± 4.85	4.21 ± 2.88	/
Follicle-stimulating hormone, IU/L	9.28 ± 4.40	8.71 ± 7.27	10.39 ± 5.83	9.22 ± 6.26	
Testosterone, ng/mL	4.16 ± 1.15	3.76 ± 0.90	4.32 ± 1.02	4.30 ± 1.00	
25-hydroxyvitamin, ng/mL	31.69 ± 11.54	29.52 ± 9.32	36.72 ± 23.82	32.11 ± 15.03	
Calcium, mmol/L	2.37 ± 0.07	2.36 ± 0.08	2.38 ± 0.10	2.37 ± 0.08	
Phosphorus, mmol/L	1.09 ± 0.12	1.04 ± 0.16	1.04 ± 0.15	1.06 ± 0.14	
Parathyroid hormone, pg/mL	45.08 ± 12.65	41.76 ± 23.41	42.84 ± 19.36	43.21 ± 18.97	
Alkaline phosphatase, U/L	70.12 ± 16.67	68.19 ± 25.04	72.50 ± 32.76	70.04 ± 24.83	
PINP, ng/mL	37.04 ± 21.28	30.83 ± 20.57	40.63 ± 34.66	36.48 ± 29.92	
β-CTX, ng/mL	0.31 ± 0.27	0.21 ± 0.18	0.31 ± 0.27	0.27 ± 0.24	
Bone mineral density (± SD)					
Lumbar spines 1–4, g/cm^2^	0.986 ± 0.113	0.878 ± 0.097^###^	0.823 ± 0.123^###^	0.903 ± 0.127	/
Femoral neck, g/cm^2^	0.766 ± 0.067	0.754 ± 0.119	0.710 ± 0.105^#^	0.750 ± 0.102	
Total hip, g/cm^2^	0.825 ± 0.066	0.769 ± 0.110^#^	0.754 ± 0.119^#^	0.785 ± 0.103	

*PINP* procollagen type I propeptides, *β-CTX* carboxyl-terminal type I collagen telopeptide

Data shown as mean or percentage as appropriate

^*^: *P* < 0.05, ^**^: *P* < 0.01, ^***^: *P* < 0.001 vs healthy controls

^#^: *P* < 0.05, ^##^: *P* < 0.01, ^###^: *P* < 0.001 vs osteopenia

^^^: *P* < 0.05, ^^^^: *P* < 0.01 vs osteoporosis

The mean age of the patients was 54.3 ± 14.4 years old. The mean serum level of T, 25(OH)D, Ca, P of the patients was 4.05 ± 1.03 ng/mL, 32.11 ± 15.03 ng/mL, 2.37 ± 0.08 mmol/L and 1.06 ± 0.14 mmol/L at baseline, respectively. Except the percentage of fragility fracture history (26.0% vs 0%, *P* < 0.001) and type 2 diabetes history (T2DM) (20.0% versus 8.0%) were higher in total patients than controls (*P* < 0.05), no differences were observed in terms of age, height, weight, and BMI between the patients and controls.

Significant differences were found between the subgroup of osteopenia and osteoporosis in age (62.7 ± 8.9 vs 45.0 ± 12.9 years old, *P* < 0.001), the percentage of T2DM (35.5% vs 12.2%, *P* < 0.05), BMD at L1-4 (0.986 ± 0.113 vs 0.878 ± 0.097 g/cm^2^, *P* < 0.001) and TH (0.825 ± 0.066 vs 0.769 ± 0.110 g/cm^2^, *P* < 0.05). Patients with severe osteoporosis had a significant higher proportion of a positive fracture history than patients with osteopenia or osteoporosis. In addition, in severe osteoporosis group, the mean BMD was 0.823 ± 0.123 g/cm^2^ at L1-4, 0.710 ± 0.105 g/cm^2^ at FN, and 0.754 ± 0.119 g/cm^2^ at TH, which were significantly lower than those of osteopenia group (*P* < 0.001 or *P* < 0.05). No significant differences in BMD were found between the severe osteoporosis and osteoporosis group. There were no significant differences in biochemical parameters among the three subgroups of men with osteopenia, osteoporosis and severe osteoporosis, including serum levels of PINP, β-CTX, Ca, P, PTH, ALP, 25OHD, LH, FSH and T.

### Baseline HRQoL of men with osteopenia/osteoporosis and its influence factors

**Fig. 2:**
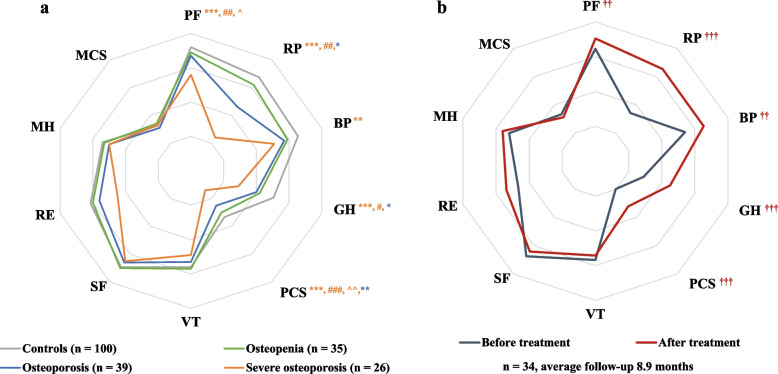
SF-36 domain scores in patients with osteoporosis and healthy controls, and its changes after bisphosphonate treatments. **a** Comparison of SF-36 domain scores among subgroups of patients with osteoporosis and healthy controls. **b** Changes in SF-36 domain scores after bisphosphonates treatment. PF, physical functioning; RP, role-physical limitation; BP, bodily pain; GH, general health; PCS, physical component summary; VT, vitality; SF, social functioning; RE, role-emotional limitation; MH, mental health; MCS, mental component summary. ^*^: *P* < 0.05, ^**^: *P* < 0.01, ^***^: *P* < 0.001 versus controls; ^#^: *P* < 0.05, ^##^: *P* < 0.01, ^###^: *P* < 0.001 versus osteopenia; ^^^: *P* < 0.05, ^^^^: *P* < 0.01 versus osteoporosis; ^††^: *P* < 0.01, ^†††^: *P* < 0.001 vs baseline

The results of baseline HRQoL were presented in Fig. 2a. There were no significant differences of baseline HRQoL scores between the patients with osteopenia and controls. In patients with osteoporosis, lower baseline HRQoL scores of RP (66.43 vs 87.34, *P* < 0.05), GH (60.63 vs 70.47, *P* < 0.05), and PCS (44.81 vs 53.19, *P* < 0.01) were found than controls. Particularly, all physical health related domains of baseline SF-36 were impaired in patients with severe osteoporosis compared to healthy controls, including PF (75.85 vs 92.03, *P* < 0.001), RP (44.00 vs 87.34,* P* < 0.01), BP (70.76 vs 85.44, *P* < 0.01), GH (49.07 vs 70.47, *P* < 0.001), and PCS (34.09 vs 53.19, *P* < 0.001). Baseline HRQoL scores were significantly lower in severe osteoporosis group than the osteopenia group in domains of PF (*P* < 0.01), RP (*P* < 0.01), GH (*P* < 0.05) and PCS (*P* < 0.001). In addition, severe osteoporosis patients had significantly lower scores in baseline PF and PCS domains than the osteoporosis group (*P* < 0.05 and *P* < 0.01, respectively). No differences were found in mental health related domains of baseline HRQoL among all subgroups of patients and the control group, including VT, SF, RE, MH and MCS. After adjusted for age, the impairment of baseline SF-36 remained in subgroup of patients with severe osteoporosis, but the difference of RP, GH, and PCS between the osteoporosis group and the control group did no longer there (Supplemental Table 1).

To investigate the possible correlated factors of baseline HRQoL in all patients with osteopenia/osteoporosis, a multiple linear regression model was conducted. Age, time since diagnosis, BMI, fragility fracture history, serum levels of T, 25(OH)D, PINP, β-CTX and BMD at L1-4 were included. The results revealed that a positive history of fragility fracture were closely correlated to a declined baseline physical health in osteopenia/osteoporosis men, including PF (β = -10.038, *P* < 0.05), RP (β = -29.590, *P* < 0.05), BP (β = -3.683, *P* < 0.05), GH (β = -15.654, *P* < 0.01) and PCS (β = -12.064, *P* < 0.01) (Table 2). None of the included factors had association with the baseline SF-36 scores of mental health (Supplemental Table 2).

**Table 2:** Factors associated with baseline quality of life in physical health domains

	**Dependent variables, β (95% CI)**				
	**PF**	**RP**	**BP**	**GH**	**PCS**
Age (year)	-0.243 (-0.532, 0.046)	-0.006 (-0.871, 0.859)	0.166 (-0.325, 0.657)	0.263 (-0.131, 0.658)	-0.051 (-0.353, 0.250)
Time since diagnosis (year)	1.290 (-0.152, 2.732)	-0.709 (-5.027, 3.609)	0.583 (-1.869, 3.036)	-1.305 (-3.275, 0.665)	0.079 (-1.427, 1.585)
BMI (kg/m^2^)	-0.485 (-1.777, 0.807)	-2.966 (-6.835, 0.902)	-1.436 (-3.633, 0.762)	-0.260 (-12.025, 1.505)	-1.012 (-2.361, 0.337)
Fragility fracture history	-10.149 (-19.220, -1.078)^*^	-29.176 (-56.344, -2.008)^*^	-3.686 (-15.279, 7.908)^*^	-12.865 (-25.260, -0.470)^**^	-12.525 (-21.998, -3.052)^**^
Testosterone level (ng/mL)	2.606 (-1.071, 6.284)	7.291 (-3.723, 18.305)	-2.503 (-8.759, 3.753)	-7.363 (-12.388, -2.338)	1.293 (-2.548, 5.133)
25-hydroxyvitamin level (ng/mL)	0.266 (-0.053, 0.585)	0.009 (-0.679, 0.698)	-0.272 (-0.663, 0.119)	-0.009 (-0.323, 0.306)	-0.053 (-0.293, 0.187)
PINP level (ng/mL)	0.149 (-0.046, 0.343)	0.220 (-0.361, 0.802)	0.093 (-0.237, 0.424)	0.143 (-0.122, 0.409)	0.123 (-0.080, 0.326)
β-CTX level (ng/mL)	-19.355 (-38.203, -0.506)	-20.022 (-76.473, 36.429)	-8.241 (-0.237, 0.424)	-15.677 (-41.341, 10.078)	-14.512 (-34.196, 5.171)
Lumbar spines 1–4 BMD (g/cm^2^)	0.866 (-0.297, 0.565)	0.578 (-0.497, 1.659)	0.478 (-0.220, 1.243)	2.142 (-4.263, 8.547)	0.783 (-1.085, 2.520)

*PF* physical functioning, *RP* role-physical limitation, *BP* bodily pain, *GH* general health, *PCS* physical component summary, *PINP* procollagen type I propeptides, *β-CTX* carboxyl-terminal type I collagen telopeptide, *BMD* bone mineral density

β, Regression coefficient. ^*^: *P* < 0.05; ^**^: *P* < 0.01

### Changes of HRQoL after bisphosphonates treatment

A prospective observation about change of HRQoL was completed in 34 men with newly diagnosed osteopenia or osteoporosis, who received ALN or ZOL treatments for 3 to 18 months (Fig. 1). The patients belonged to severe osteoporosis (*n* = 4), osteoporosis (*n* = 19) and osteopenia (*n* = 11) group respectively, of which 22 patients received ALN treatment, and 12 patients received ZOL treatment. After treatments of ALN or ZOL, all scales of physical health of HRQoL were improved, including PF (84.63 to 90.53, *P* < 0.01), RP (54.21 to 85.53,* P* < 0.001), BP (74.11 to 85.32, *P* < 0.01), GH (49.05 to 65.03, *P* < 0.001), and PCS (39.78 to 52.05, *P* < 0.001). Changes in domain scores of SF-36 after bisphosphonates treatment were shown in Fig. 2b. For mental health, no significant change was observed after ALN or ZOL treatment.

**Fig. 3:**
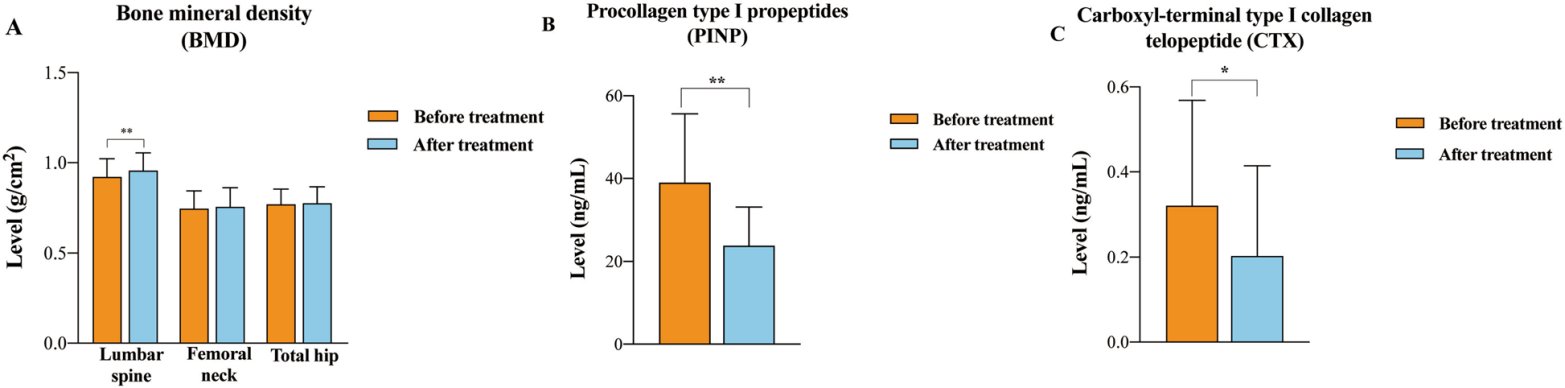
Changes of BMD and bone turnover markers before and after bisphosphonates treatment. **a** Changes in BMD before and after bisphosphonates treatment. **b** Changes in the serum level of PINP before and after bisphosphonates treatment. **c** Changes in the serum level of β-CTX before and after bisphosphonates treatment. Data were shown as mean and standard deviation. *: *P* < 0.05, **: *P* < 0.01, ***: *P* < 0.001 after vs baseline

As shown in Fig. 3, after an average follow-up time of 8.9 months, the areal BMD at L1-4 increased from 0.921 ± 0.102 to 0.956 ± 0.099 g/cm^2^ after treatments (*P* < 0.001), meanwhile, the areal BMD at FN and TH had increasing trend, but did not reach significant differences (Fig. 3a). The serum levels of PINP (39.04 ± 17.19 ng/mL to 23.20 ± 9.19 ng/mL, *P* < 0.01) and β-CTX (0.33 ± 0.25 to 0.20 ± 0.18 ng/mL, *P* < 0.05) significantly decreased after ALN or ZOL treatment (Fig. 3b, c).

To assess the possible correlated factors of improvements in HRQoL after bisphosphonates treatment, the linear regressions was completed between respective changes of BMD or bone metabolic markers and changes of HRQoL scores, which adjusted for age and fragility fracture history (Supplemental tables 3 and 4). The decrease of β-CTX level had a positive correlation with the improvement in PF scores (β = 21.807, *P* < 0.05), and the increase of TH BMD had a positive correlation with the change of VT scores (β = 784.314, *P* < 0.01). Changes of BMD in LS and FN, and the serum levels of Ca, P, PTH, 25(OH)D, ALP or PINP had no association with the improvement in HRQoL after bisphosphonates treatment.

## Discussion

In this study, we performed a detailed investigation about the HRQoL of men with primary osteoporosis, and evaluated its influencing factors. The results indicated that HRQoL of the physical health domain was significantly impaired in men with osteopenia and osteoporosis, especially in men with severe osteoporosis. We found that positive history of fragility fracture was closely correlated with lower HRQoL scores. Moreover, we found for the first time that the treatment of bisphosphonates could improve scores of physical health domains, including PF, RP, BP, GH, and PCS of Chinese men with osteoporosis.

Osteoporosis can lead to multiple adverse consequences, including chronic pain, fragility fractures, sarcopenia, physical disability, which may impair the quality of life of the patients [28, 29]. We evaluated the HRQoL of Chinese men with primary osteoporosis or osteopenia in detail for the first time, and confirmed that the poorer quality of life in osteoporotic men than healthy controls, especially in the physical function domain. Two meta-analysis studies demonstrated that HRQoL of men with osteoporosis was impaired more obviously in physical function than mental function, which were consistent with our results [12, 14, 30]. Through EQ-5D questionnaire, another study found that pain/discomfort and anxiety/depression were common in men with osteoporosis [28].

Furthermore, we found that a positive history of fragility fracture was significantly correlated with impaired HRQoL of men with osteoporosis or osteopenia, even after adjusting for age, BMI, BMD, serum levels of 25(OH)D and testosterone. These results were consistent with previous studies [31, 32]. In a 10-year longitudinal assessment of HRQoL in self-reported osteoporosis patients (2797 women and 1023 men), patients with fragility fracture showed a greater decline in PF, RP, BP, and PCS domains than patients without fractures [29]. Bone fracture would lead to pain, skeletal deformities, difficulty in movement, and other adverse consequences, which would significantly impair HRQoL of patients [33, 34]. In addition, men with fragility fractures usually had a significant loss of muscle mass and strength, which would lead to physical dysfunction and high risk of fall, and then increased the risk of refracture [35, 36]. Taken together, osteoporotic fractures can cause multiple adverse consequences and reduce HRQoL of patients.

In addition to fragility fracture, decreased BMD was found to be a negative factor of HRQoL in osteoporosis patients. *Cooper *et al*.* found a lower femoral BMD T-score was associated with poorer HRQoL scores in PF, SF, and GH domains of men with osteoporosis [12]. Another study included 62 old men, and found a lower BMD at distal radius was correlated to impaired quality of life [37]. A similar correlation was also observed between lumbar BMD and HRQoL in osteoporosis men [38]. However, there were other studies implied that BMD had no significant association with quality of life. The OFELY study reported that scores on the physical difficulty domain of a cohort of 756 women did not differ according to BMD [39]. Our results indicated that there was no significant association between BMD and HRQoL scores of osteopenia/osteoporosis patients. Since a majority of fragility fractures occurred in patients whose BMD did not reach osteoporosis [40], these results might imply fragility fracture might be a more important influencing factor of the HRQoL other than BMD. Moreover, multiple factors increased the risk of osteoporosis in men, including hypogonadism, vitamin D deficiency, low BMI, alcohol abuse and cigarette addiction [41, 42], and whether they impaired quality of life is worth investigation.

As we know, bisphosphates are widely used for treatment of male osteoporosis [43], which could increase BMD, decrease bone turnover markers and reduce bone fracture risks of men with osteoporosis [44, 45]. The effects of bisphosphonates treatment could improve the HRQoL of women with osteoporosis [46], but few studies evaluated the influence of anti-osteoporotic drugs on life quality of men with osteoporosis. Our study found for the first time that treatment with alendronate and zoledronic acid could significantly improve the life quality of men with primary osteoporosis, especially in domains of PF, RP, BP, GH, and PCS. However, studies with larger sample and longer follow-up period were needed to confirm this result. In addition, denosumab and teriparatide were demonstrated to increase BMD and reduce fracture risk of men with osteoporosis [19, 47]. Only a study showed that denosumab and alendronate could significantly increase BMD and improve the PCS and MCS of HRQoL of men with non-metastatic prostate cancer receiving androgen deprivation therapy [48]. The effects of a variety of anti-osteoporotic agents on quality of life of men with osteoporosis were still worthy further studies.

In this study, we identified the impaired life quality and its correlation factors for the first time in a cohort of Chinese men with primary osteoporosis. We demonstrated that alendronate and zoledronic acid treatment could significantly improve HRQoL of osteopenia/osteoporosis men. However, there were several limitations in this study. Firstly, the sample size was relatively small, and the BMD and bone turnover biomarkers were not available in healthy controls. Secondly, the patients receiving bisphosphonates treatment were few and the follow-up time was relatively short, and it was difficult to compare the effects of oral or intravenous bisphosphonates on HRQoL of men with osteoporosis. Finally, we did not collect detailed data of lifestyle (e.g. caffeine, tea), dietary intake, and physical activity at the baseline, therefore we could not rule out the impact of these factors on HRQoL.

## Conclusion

Osteoporosis can significantly impair the quality of life of men with primary osteoporosis, especially in physical function domain, and the more severe the osteoporosis, the lower the quality of life. Positive history of fragility fractures is the most important relevant factor for the impairment of HRQoL. Treatments of alendronate or zoledronic acid are beneficial to improve the quality of life of men with osteoporosis. The effects of a variety of anti-osteoporotic agents on the quality of life of male patients with osteoporosis needs to be further studied.

## Supplementary Information

**Additional file 1:** **Supplemental table 1.** SF-36 domain scores adjusted for age in patients with osteoporosis and controls. **Supplemental table 2.** Factors associated with baseline quality of life in mental health domains. **Supplemental table 3.** Factors associated with changes in physical health domain of quality of life after bisphosphonates treatment. **Supplemental table 4.** Factors associated with changes in mental health domain of quality of life after bisphosphonates treatment.

**Additional file 2: Supplemental figure. **Changes in bone metabolic markers after bisphosphonates treatment.

## Data Availability

All data generated or analyzed during this study are included in this published article and its supplementary information files.
