# Supplementary material for: Health-related quality of life of men with primary osteoporosis and its changes after bisphosphonates treatment
Source: BMC Musculoskelet Disord. 2023 Apr 19;24:309. doi: 10.1186/s12891-023-06397-8 (PMC10114430; doi:10.1186/s12891-023-06397-8)
Supplement: Supplementary file 1 — Additional file 1: Supplemental table 1. SF-36 domain scores adjusted for age in patients with osteoporosis and controls. Supplemental table 2. Factors associated with baseline quality of life in mental health domains. Supplemental table 3. Factors associated with changes in physical health domain of quality of life after bisphosphonates treatment. Supplemental table 4. Factors associated with changes in mental health domain of quality of life after bisphosphonates treatment. [file 12891_2023_6397_MOESM1_ESM.pptx]

## Slide 1
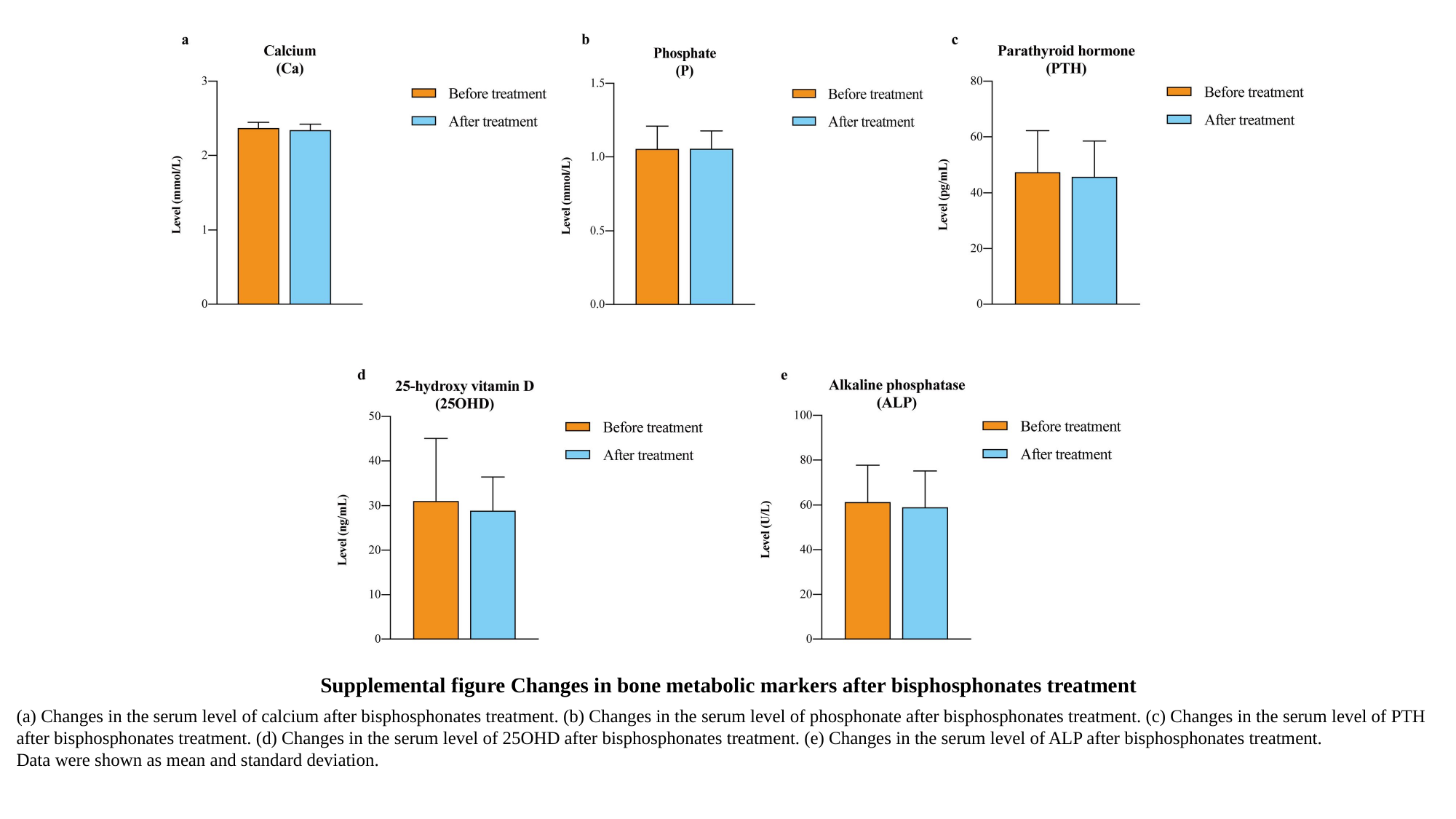

Supplemental figure Changes in bone metabolic markers after bisphosphonates treatment
(a) Changes in the serum level of calcium after bisphosphonates treatment. (b) Changes in the serum level of phosphonate after bisphosphonates treatment. (c) Changes in the serum level of PTH after bisphosphonates treatment. (d) Changes in the serum level of 25OHD after bisphosphonates treatment. (e) Changes in the serum level of ALP after bisphosphonates treatment.
Data were shown as mean and standard deviation.
