# Supplementary material for: Health-related quality of life of men with primary osteoporosis and its changes after bisphosphonates treatment
Source: BMC Musculoskelet Disord. 2023 Apr 19;24:309. doi: 10.1186/s12891-023-06397-8 (PMC10114430; doi:10.1186/s12891-023-06397-8)
Supplement: Supplementary file 2 — Additional file 2: Supplemental figure. Changes in bone metabolic markers after bisphosphonates treatment. [file 12891_2023_6397_MOESM2_ESM.docx]

**Supplemental table 1.** **SF-36 domain scores adjusted for age in patients with osteoporosis and controls.**

|  | **Osteopenia (n = 35)** | **Osteoporosis (n = 39)** | **Severe osteoporosis (n = 26)** | **Controls (n = 100)** | **Adjusted *P*-value** |
| --- | --- | --- | --- | --- | --- |
| Physical functioning | 89.10 ± 11.05 | 87.43 ± 12.05 | 75.85 ± 16.99^***, ##, ^^ | 92.03 ± 12.90 | <0.001 |
| Role-physical limitation | 82.44 ± 33.91 | 66.43 ± 39.73 | 44.00 ± 40.80^***, ##^ | 87.34 ± 28.83 | <0.001 |
| Bodily pain | 79.29 ± 20.75 | 77.38 ± 22.16 | 70.76 ± 21.97^**^ | 85.44 ± 17.93 | 0.009 |
| General health | 62.29 ± 13.99 | 60.63 ± 22.58 | 49.07 ± 16.10^***^ | 70.47 ± 18.12 | <0.001 |
| Physical component summary | 50.22 ± 10.97 | 44.81 ± 13.62 | 34.09 ± 15.37^***, ###, ^^^ | 53.19 ± 10.74 | <0.001 |
| Vitality | 77.29 ± 14.31 | 72.69 ± 16.58 | 68.65 ± 18.21 | 76.01 ± 15.30 | 0.102 |
| Social functioning | 89.52 ± 13.19 | 85.76 ± 16.31 | 85.22 ± 20.47 | 89.17 ± 14.34 | 0.510 |
| Role-emotional limitation | 80.31 ± 36.26 | 76.35 ± 34.12 | 65.33 ± 37.86 | 81.43 ± 34.49 | 0.295 |
| Mental health | 73.37 ± 14.42 | 70.26 ± 14.96 | 70.21 ± 18.07 | 73.77 ± 14.97 | 0.353 |
| Mental component summary | 54.46 ± 8.43 | 50.89 ± 9.74 | 53.06 ± 8.48 | 52.57 ± 8.17 | 0.734 |

^**^: *P* < 0.01, ^***^: *P* < 0.001 vs controls; ^##^: *P* < 0.01, ^###^: *P* < 0.001 vs osteopenia; ^^^: *P* < 0.05, ^^^^: *P* < 0.01 vs osteoporosis.

**Supplemental table 2.** **Factors associated with baseline quality of life in mental health domains.**

|  | **Dependent variables, β (95% CI)** | | | | |
| --- | --- | --- | --- | --- | --- |
|  | **VT** | **SF** | **RE** | **MH** | **MCS** |
| Age (year) | 0.028 (-0.362, 0.419) | -0.039 (-0.451, 0.373) | -0.068 (-0.898, 0.761) | 0.152 (-0.190, 0.495) | 0.062 (-0.164, 0.289) |
| Time since diagnosis (year) | -0.660 (-2.611, 1.290) | 0.645 (-1.412, 2.702) | 1.590 (-5.734, 2.555) | 0.448 (-1.264, 2.160) | -0.200 (-1.331, 0.931) |
| BMI (kg/m^2^) | -0.064 (-1.811, 1.684) | 0.132 (-1.711, 1.975) | 0.950 (-2.763, 4.663) | -0.033 (-1.567, 1.500) | 0.327 (-0.686, 1.341) |
| Fragility fracture history | -12.393 (-24.667, -0.120) | 5.463 (-7.478, 18.404) | -17.727 (-43.806, 8.351) | -0.953 (-11.724, 9.818) | 0.389 (-6.726, 7.505) |
| Testosterone level (ng/mL) | -3.498 (-8.474, 1.477) | 2.662 (-2.584, 7.909) | 5.984 (-4.588, 16.566) | -2.805 (-7.172, 1.561) | -0.502 (-3.387, 2.382) |
| 25-hydroxyvitamin level (ng/mL) | 0.098 (-0.213, 0.409) | -0.290 (-0.618, 0.038) | 0.305 (-0.356, 0.965) | -0.030 (-0.303, 0.243) | 0.011 (-0.170, 0.191) |
| PINP level (ng/mL) | 0.030 (-0.233, 0.293) | -0.044 (-0.321, 0.233) | 0.288 (-0.270, 0.846) | -0.049 (-0.280, 0.181) | -0.022 (-0.175, 0.130) |
| β-CTX level (ng/mL) | 3.449 (-22.053, 28.591) | -5.004 (-31.893, 21.885) | 5.551 (-48.634, 59.737) | 0.847 (-21.534, 23.227) | 4.914 (-9.870, 19.699) |
| Lumbar spines 1-4 BMD (g/cm^2^) | 0.928 (-44.013, 45.868) | -14.921 (-62.306, 32.463) | -73.756 (-169.242, 21.731) | 15.601 (-55.040, 23.838) | -6.329 (-32.383, 19.724) |

VT, vitality; SF, social functioning; RE, role-emotional limitation; MH, mental health; MCS, mental component summary; PINP, procollagen type I propeptides; β-CTX, carboxyl-terminal type I collagen telopeptide; BMD, bone mineral density.

**Supplemental table 3. Factors associated with changes in physical health domain of quality of life after bisphosphonates treatment.**

|  | **Changes of PF** | **Changes of RP** | **Changes of BP** | **Changes of GH** | **Changes of PCS** |
| --- | --- | --- | --- | --- | --- |
| Changes in LS BMD (g/cm^2^) | 109.855 (-7.449, 227.160) | -1.059 (-2.249, 0.130) | -120.363 (-419.969, 177.697) | 55.716 (-231.661, 343.094) | -84.438 (-255.076, 40.670) |
| Changes in FN BMD (g/cm^2^) | -7.014 (-145.359, 131.332) | -125.867 (-879.067, 627.333) | -118.481 (-405.593, 168.630) | -64.590 (-340.322, 211.141) | -25.289 (-0.515, 0.060) |
| Changes in TH BMD (g/cm^2^) | 122.624 (-91.051, 336.299) | 123.934 (-1149.301, 1397.169) | -405.727 (-808.342, -3.112) | -149.555 (-606.723, 307.613) | -24.171 (-318.891, 270.549) |
| Changes in Ca level (mmol/L) | -4.953 (-73.601, 63.695) | 136.251 (-105.442, 377.944) | -63.549 (-175.643, 48.546) | -46.539 (-114.166, 21.449) | 37.224 (-21.940, 96.388) |
| Changes in P level (mmol/L) | -4.583 (-36.567, 27.401) | 49.268 (-65.755, 164.290) | -31.309 (-83.368, 20.749) | -4.759 (-38.490, 28.972) | 6.586 (-22.427, 35.598) |
| Changes in PTH level (pg/mL) | -0.011 (-0.319, 0.296) | -0.415 (-1.494, 0.655) | 0.215 (-0.284, 0.713) | -0.089 (-0.407, 0.229) | -0.093 (-0.361, 0.175) |
| Changes in 25(OH)D level (ng/mL) | 0.197 (-0.151, 0.545) | -0.293 (-1.664, 1.078) | 0.200 (-0.422, 0.821) | 0.147 (-0.233, 0.527) | 0.064 (-0.272, 0.399) |
| Changes in ALP level (U/L) | -0.324 (-0.836, 0.188) | -0.313 (-2.176, 1.551) | -0.109 (-1.063, 0.844) | 0.162 (-0.332, 0.656) | -0.129 (-0.554, 0.295) |
| Changes in P1NP level (ng/mL) | -0.159 (-0.452, 0.134) | 0.117 (-0.892, 1.127) | -0.004 (-0.518, 0.509) | -0.085 (-0.403, 0.234) | -0.057 (-0.321, 0.208) |
| Changes in β-CTX level (ng/mL) | 21.807 (40.938, 2.677)^*^ | 38.539 (121.998, -44.919) | 22.325 (59.694, -15.043) | 6.069 (30.133, -17.996) | 11.243 (31.311, -8.826) |

PF, physical functioning; RP, role-physical limitation; BP, bodily pain; GH, general health; PCS, physical component summary; BMD, bone mineral density; LS, lumbar spines 1-4; FN, femoral neck; TH, total hip; Ca, calcium; P, phosphorus; PTH, parathyroid hormone; 25(OH)D, 25-hydroxyvitamin; ALP, alkaline phosphatase; PINP, procollagen type I propeptides; β-CTX, carboxyl-terminal type I collagen telopeptide.

β, Regression coefficient. Adjusted for age, and fragility fracture history.

^*^: *P* < 0.05.

**Supplemental table 4. Factors associated with changes in mental health domain of quality of life after bisphosphonates treatment.**

|  | **Changes of VT** | **Changes of SF** | **Changes of RE** | **Changes of MH** | **Changes of MCS** |
| --- | --- | --- | --- | --- | --- |
| Changes in LS BMD (g/cm^2^) | 258.832 (-181.132, 698.797) | -379.046 (-931.145, 173.052) | 792,174 (-288.564, 1872.912) | 40.020 (-338.185, 418.224) | 67.196 (-206.044, 340.437) |
| Changes in FN BMD (g/cm^2^) | -9.469 (-473.438, 454.500) | -391.573 (-913.163, 130.018) | -123,217 (-1310.064, 1063.629) | 68.890 (-293.182, 430.961) | -68.489 (-331.381, 194.403) |
| Changes in TH BMD (g/cm^2^) | 784.314 (275.234, 1293.394)^**^ | 254.028 (-735.546, 1243.603) | 1043,279 (-797.687, 2884.245) | 339.977 (-219.181, 899.134) | 336.830 (-35.309, 708.968) |
| Changes in Ca level (mmol/L) | -23.976 (-154.012, 106.059) | -48.057 (-198.870, 102.755) | -89.643 (-387.187, 207.900) | 15.174 (-82.132, 112.480) | -22.623 (-86.701, 41.455) |
| Changes in P level (mmol/L) | 24.146 (-35.427, 83.719) | 21.058 (-49.499, 91.616) | 80.015 (-53.772, 213.801) | 23.501 (-20.235, 67.237) | 21.778 (-6.250, 49.806) |
| Changes in PTH level (pg/mL) | -0.354 (-0.852, 0.143) | 0.015 (-0.648, 0.679) | -0.658 (-1.959, 0.643) | -0.269 (-0.675, 0.137) | -0.169 (-0.439, 0.101) |
| Changes in 25(OH)D level (ng/mL) | -0.524 (-1.146, 0.098) | 0.079 (-0.695, 0.854) | -0.270 (-1.909, 1.369) | -0.238 (-0.749, 0.272) | -0.175 (-0.507, -0.156) |
| Changes in ALP level (U/L) | -0.231 (-1.268, 0.806) | 0.956 (-0.139, 2.051) | -0.869 (-3.178, 1.440) | -0.225 (-0.990, 0.539) | 0.062 (-0.449, 0.573) |
| Changes in P1NP level (ng/mL) | 0.053 (-0.480, 0.586) | 0.310 (-0.306, 0.926) | 0.225 (-1.143, 1.592) | -0.051 (-0.444, 0.341) | 0.095 (-0.164, 0.354) |
| Changes in β-CTX level (ng/mL) | 18.696 (60.512, -23.120) | 21.108 (68.071, -25.856) | 69.072 (164.038, -25.893) | 13.775 (45.827, -18.277) | 13.467 (33.699, -6.765) |

VT, vitality; SF, social functioning; RE, role-emotional limitation; MH, mental health; MCS, mental component summary; P BMD, bone mineral density; LS, lumbar spines 1-4; FN, femoral neck; TH, total hip; Ca, calcium; P, phosphorus; PTH, parathyroid hormone; 25(OH)D, 25-hydroxyvitamin; ALP, alkaline phosphatase; PINP, procollagen type I propeptides; β-CTX, carboxyl-terminal type I collagen telopeptide.

β, Regression coefficient. Adjusted for age, and fragility fracture history.

^**^: *P* < 0.01.
